# Quantitative phosphoproteomic analysis identifies the potential therapeutic target EphA2 for overcoming sorafenib resistance in hepatocellular carcinoma cells

**DOI:** 10.1038/s12276-020-0404-2

**Published:** 2020-03-19

**Authors:** Chih-Ta Chen, Li-Zhu Liao, Ching-Hui Lu, Yung-Hsuan Huang, Yu-Kie Lin, Jung-Hsin Lin, Lu-Ping Chow

**Affiliations:** 1 Graduate Institute of Biochemistry and Molecular Biology, College of Medicine, National Taiwan University, Taipei, Taiwan; 2 Research Center for Applied Sciences, Academic Sinica, Taipei, Taiwan

**Keywords:** Cancer therapeutic resistance, Protein-protein interaction networks

## Abstract

Limited therapeutic options are available for advanced-stage hepatocellular carcinoma owing to its poor diagnosis. Drug resistance to sorafenib, the only available targeted agent, is commonly reported. The comprehensive elucidation of the mechanisms underlying sorafenib resistance may thus aid in the development of more efficacious therapeutic agents. To clarify the signaling changes contributing to resistance, we applied quantitative phosphoproteomics to analyze the differential phosphorylation changes between parental and sorafenib-resistant HuH-7 cells. Consequently, an average of ~1500 differential phosphoproteins were identified and quantified, among which 533 were significantly upregulated in resistant cells. Further bioinformatic integration via functional categorization annotation, pathway enrichment and interaction linkage analysis led to the discovery of alterations in pathways associated with cell adhesion and motility, cell survival and cell growth and the identification of a novel target, EphA2, in resistant HuH-7^R^ cells. In vitro functional analysis indicated that the suppression of EphA2 function impairs cell proliferation and motility and, most importantly, overcomes sorafenib resistance. The attenuation of sorafenib resistance may be achieved prior to its development through the modulation of EphA2 and the subsequent inhibition of Akt activity. Binding analyses and in silico modeling revealed a ligand mimic lead compound, prazosin, that could abate the ligand-independent oncogenic activity of EphA2. Finally, data obtained from in vivo animal models verified that the simultaneous inhibition of EphA2 with sorafenib treatment can effectively overcome sorafenib resistance and extend the projected survival of resistant tumor-bearing mice. Thus our findings regarding the targeting of EphA2 may provide an effective approach for overcoming sorafenib resistance and may contribute to the management of advanced hepatocellular carcinoma.

## Liver cancer: A new route to fighting drug resistance

Inhibiting the activity of a protein known as EphA2 may help overcome the development of drug resistance during treatment of hepatocellular carcinoma, the most common form of liver cancer. Resistance often develops to sorafenib, the main drug treatment option for the advanced disease. Researchers in Taiwan led by Lu-Ping Chow at the National Taiwan University in Taipei investigated the role of around 1500 phosphoproteins, which are regulated by the attachment and removal of small chemical groups containing phosphorus and oxygen. The activity of the phosphoprotein EphA2 was identified as a major contributor to drug resistance. This was linked to the ability of EphA2 to interfere with a molecular signaling pathway previously implicated in sorafenib activity. Suppressing EphA2 activity suppressed the development of sorafenib resistance in cultured cells and in mice with liver cancer, suggesting therapeutic possibilities.

## Introduction

Hepatocellular carcinoma (HCC) is one of the most common cancer types worldwide and is responsible for approximately 600,000 deaths each year^1,2^. However, the majority of HCC patients are diagnosed with advanced-stage tumors requiring systemic therapy^3,4^. In view of the limited progress in systemic therapy, the development of novel treatment options for HCC, especially in advanced stages, remains critical for human health^5^. Sorafenib, a multikinase inhibitor mainly targeting vascular endothelial growth factor receptor, platelet-derived growth factor receptor beta, and Raf kinases, prolongs the overall survival of advanced HCC patients by 6–9 months and is currently the only effective targeted therapy approved by the Food and Drug Administration^6,7^. Despite this significant improvement in survival, the efficacy of sorafenib against HCC is modest, with an objective tumor response rate as low as 2–3%^2^. The majority of patients showing a significant initial response to sorafenib eventually develop progressive disease and drug resistance, which remains a major obstacle in the successful treatment of advanced HCC.

Previous studies have indicated that acquired/secondary resistance results from conditions that develop during sorafenib treatment. Several possible mechanisms of sorafenib resistance have been proposed, including epidermal growth factor receptor (EGFR) activation, Akt activation, c-Jun activation, induction of hypoxia, autophagy, apoptosis, cancer stem cell renewal, and epithelial–mesenchymal transition activation^8^. For instance, the effect of sorafenib on growth inhibition is impaired in HCC cells showing a higher level of EGFR activation, and either the downregulation of the expression or the inhibition of the kinase activity of EGFR contributes to increasing sensitivity to sorafenib^9^. Some studies have shown that the phosphoinositide 3-kinase (PI3)K/Akt pathway is activated as a mechanism to overcome sorafenib-induced cell death^10^. It has also been proposed that HCC cells exhibiting sorafenib resistance present higher metastatic potential^11^. Given the limited current therapeutic options for progressive HCC and the uncertain application of the proposed models, there is still an urgent need to elucidate the molecular pathways involved in the development of drug resistance and identify effective targets that could provide benefits to patients beyond sorafenib therapy.

Protein phosphorylation plays an important role in cellular regulation in eukaryotic cells. The dysregulation of specific signaling pathways and mutation of critical kinases are frequently associated with cancer development^12^. The elucidation of the mechanisms underlying signaling aberrations in cancers may therefore aid in the identification of the cellular processes involved in tumor progression and potential therapeutic targets. In recent years, mass spectrometry (MS)-based phosphoproteomics has provided an invaluable tool for the comprehensive discovery of signaling networks involved in drug resistance. Following the treatment of B-RAF mutant melanoma with different mitogen-activated protein kinase (MAPK) pathway inhibitors, a stable isotope labeling by amino acids in cell culture (SILAC)-based phosphoproteomic analysis revealed the inhibitor-specific regulation of MAPK kinase 1/2 or extracellular signal-regulated kinase 1/2 and an off-target reaction with p38α and led to the identification of several novel targets in the B-RAF-driven pathway^13^. Similarly, quantitative phosphoproteomic analysis of secondary resistant gastrointestinal stroma tumors helped to identify several bypassing KIT downstream elements and the simultaneous activation of EGFR^14^. Several phosphoproteomic studies have been successfully implemented, so we utilized a phosphoproteomic approach to investigate the molecular alterations during the development of sorafenib resistance.

In this study, we adopted an MS-based quantitative phosphoproteomic approach to delineate the mechanisms underlying acquired resistance against sorafenib in HCC. We observed a highly activated interconnected network in sorafenib-resistant cells, within which a novel receptor tyrosine kinase (RTK), ephrin type-A receptor 2 (EphA2), was identified as a potential target for the modulation of this dysregulated downstream network. Subsequent in vitro functional assays showed that the suppression of EphA2 expression impairs proliferation, tumorigenicity, and motility in resistant HCC cells. A lead compound, prazosin, was identified through targeted small-scale drug screening and characterized as a novel agonistic inhibitor of EphA2. Prazosin inhibited cell proliferation and migration, induced apoptosis, and increased sorafenib sensitivity in HuH-7^R^ cells, possibly by inhibiting Akt activity. Combined treatment with prazosin and sorafenib synergistically suppressed tumor growth and prolonged overall survival in vivo. In this study, we focused on elucidating the molecular mechanisms contributing to sorafenib resistance, and we present a novel therapeutic approach for the treatment of advanced HCC patients by targeting EphA2.

## Materials and methods

### Cell culture, EphA2 knockdown, EphA2 mutant expression, and functional assays

The HCC cell line HuH-7 was obtained from the Health Science Research Resources Bank (JCRB0403). A resistant HCC cell line with acquired sorafenib resistance, HuH-7^R^, was established from HuH-7 cells as described previously^15^. PLC-5, Sk-Hep-1 and Hep3B cells were obtained from the American Type Culture Collection. HuH-7, HuH-7^R^, PLC-5, Sk-Hep-1, Hep3B, and HEK-293T cells were maintained in Dulbecco’s modified Eagle’s medium (DMEM; HyClone) supplemented with 10% fetal bovine serum, penicillin (100 U/L), and streptomycin (10 mg/L) in a 37 °C humidified incubator under 5% CO_2_. The target sequences for EphA2 knockdown were TCGGACAGACATATAGGATAT (shEphA2#1) and CCATCAAGATGCAGCAGTATA (shEphA2#2). Lentiviruses expressing a small hairpin RNA (shRNA) targeting EphA2 (shEphA2#1 and #2) or a control shRNA (shCtrl) were produced in HEK293T cells. Medium containing the shEphA2 or shCtrl viruses was added to HuH-7^R^ or Sk-Hep-1 cell cultures. Cell proliferation, colony formation, viability, wound healing, and invasion assays were performed after the establishment of knockdown. The EphA2 gene was cloned from a cDNA library generated from HuH-7^R^ cells; the EphA2 S897A mutant was constructed via site-directed mutagenesis by PCR as described previously^16^. Both wild-type and mutant EphA2 were cloned into the pLAS2w.Ppuro lentivirus expression vector. Lentiviruses expressing mutant (S897A) or wild-type EphA2 were produced in HEK293T cells. Media containing the mutant or wild-type viruses were added to HuH-7^R^ cell cultures. Functional assays were performed as described above after confirmation of the expression by western blotting.

### SILAC sample preparation, fractionation, and phosphopeptide enrichment

For SILAC, HuH-7^R^ cells were heavily labeled with [^13^C_6_]-L-lysine and [^13^C_6_, ^15^N_4_]-L-arginine in DMEM (Life Sciences). HuH-7 cells were maintained in the same medium containing unlabeled amino acids. Labeled HuH-7 and HuH-7^R^ cells were lysed in cell extraction buffer containing 8 M urea, 10 mM dithiothreitol (DTT), a phosphatase inhibitor (Sigma-Aldrich), and a protease inhibitor cocktail (Pierce) (pH 7.0). Subsequently, the lysates were reduced with DTT and alkylated with iodoacetamide at 37 °C. Equal amounts of light and heavy samples were mixed. The combined SILAC-labeled lysates were diluted 10× and digested with trypsin (1:50, w/w) (Promega) in 50 mM ammonium bicarbonate at 37 °C overnight. The digested samples were desalted with a C18 cartridge (J.T. Baker) as suggested by the manufacturer and reconstituted in 0.1% trifluoroacetic acid binding buffer. The desalted and digested samples were then fractionated into 10 parts via a gradient of 5–50% acetonitrile (ACN) in TEA buffer with a High pH Reversed-Phase Peptide Fractionation Kit (Thermo Scientific) according to the manufacturer’s instructions. The fractionated peptides were dried and reconstituted in phosphopeptide binding buffer containing 30% lactic acid, 40% ACN, and 4% formic acid. A self-packed TiO_2_ tip column was activated with methanol and balanced with binding buffer. The digested SILAC samples were applied to the column for phosphopeptide enrichment. The enriched phosphopeptides were sequentially eluted with 200 mM ammonium bicarbonate/water, pH 9.0, and 0.5% piperidine/water, pH 11.0, and were neutralized with 20% formic acid/water^17^. The eluted peptides were dried with a SpeedVac (Heto).

### Liquid chromatography tandem MS analyses

Enriched SILAC samples were analyzed separately with an LTQ-Orbitrap Velos mass analyzer (Thermo Fisher Scientific). Each sample was reconstituted in 2% ACN and 0.1% formic acid/water, injected into a reverse-phase C18 trap column (Acclaim PepMap100; Thermo Fisher Scientific), and separated in a coupled reverse-phase C18 chromatography column (Acclaim PepMap RSLC; Thermo Fisher Scientific). The chromatography gradient program was set as follows: increase in buffer B from 4% to 30% within 100 min for peptide separation. The mobile phases consisted of buffer A (0.1% formic acid/water) and buffer B (90% ACN in 0.1% formic acid/water). The analysis parameters were set as described previously^17^. The neutral loss mode detection of phosphorylation sites was included with *m*/*z* values of 24.5, 33.3, 49, and 98 for the top 5 ions.

### Phosphoprotein identification and quantification

For phosphoprotein identification and quantification, the two raw spectrum files were processed and quantified as a single event using the Proteome Discoverer software (Version 1.3; Thermo Fisher Scientific) with the Mascot search engine (version 2.3.02) against the *Homo sapiens* protein database containing 20,232 entries (Swiss-Prot 57.2 version). The criteria for identification and SILAC-based quantification were set as described previously with static modifications of deamidation (NQ), oxidation (M), and N-terminal acetylation, additionally including phosphoserine, phosphothreonine, and phosphotyrosine^18^. The enzyme specificity was set to trypsin with a maximum of two missed cleavages. The precursor mass tolerance was set at 10 ppm, and the fragment ion mass tolerance was set to 0.5 Da. False discovery rate (FDR) was calculated by enabling peptide sequence analysis using a decoy database. The identified peptides were validated using a Percolator algorithm with an FDR threshold of 0.01.

### Bioinformatic analysis

Phosphoproteins with at least one quantified phosphorylation site showing a >1.5-fold increase (H/L ≥ 1.5) in at least two replicates of three independent experiments were subjected to clustered functional enrichment analyses with DAVID (Database for Annotation, Visualization and Integrated Discovery; https://david.ncifcrf.gov/home.jsp). In the clustered functional enrichment analysis, upregulated phosphoproteins (H/L ≥ 1.5) were enriched according to the molecular function, cellular compartment, and biological process categories and were further categorized into related clusters. To investigate the cellular pathways involved, the upregulated (H/L ≥ 1.5) phosphoproteins were also subjected to pathway enrichment analysis by using DAVID based on the Kyoto Encyclopedia of Genes and Genomes (KEGG) pathway database. For the elucidation of the interaction network, nonredundant molecules in the significantly enriched pathways were selected and subjected to STRING analysis. The correlation confidence was intermediate (≥0.400), and interaction construction was based on text mining, experiments, and databases.

### Western blotting, immunofluorescent staining, and immunohistochemistry

HuH-7, HuH-7^R^ and Sk-Hep-1 cells were subjected to lentivirus-mediated knockdown, EphA2 (wild-type and mutant) expression, or treatment with Ephrin-A1-Fc (R&D) or prazosin (Sigma-Aldrich). All samples were lysed in lysis buffer containing 50 mM Tris-HCl, 150 mM NaCl, 1 mM EDTA, 1% Triton X-100, a 1× phosphatase inhibitor, and a 1× protease inhibitor cocktail (pH 7.4). The lysates were separated via sodium dodecyl sulfate-polyacrylamide gel electrophoresis (SDS-PAGE) and transferred to polyvinylidene difluoride membranes. Antibodies for western blotting against EphA2, p-EphA2 Y772, p-EphA2 S897, cleaved caspase 3, and cleaved poly ADP-ribose polymerase (PARP) were acquired from Cell Signaling Technology, and an actin antibody was acquired from Millipore. Western blot analyses were conducted as described previously^19^. The ligand-dependent EphA2 internalization induced by prazosin was examined via immunofluorescent staining with a specific anti-EphA2 antibody, as described previously^16^. Briefly, HuH-7^R^ cells were seeded on coverslips overnight and then treated with 10 μM prazosin for 0, 2, and 4 h at 37 °C. After treatment, the cells were fixed, blocked, and immunostained with an antibody against EphA2 followed by a tetramethylrhodamine-isothiocyanate (TRITC)-labeled secondary antibody. Nuclear staining was performed with 4,6-diamidino-2-phenylindole (DAPI), and images were captured using a Carl Zeiss LSM880 confocal microscope (Zeiss, Jena, Germany). Apoptosis induced by EphA2 inhibition was analyzed via Hoechst 33342 staining^20^. The percentage of apoptotic cells in the groups treated with 10 μM prazosin for 0, 2, 4, or 6 h was determined by counting the cells showing nuclear fragmentation. Xenograft tumors harvested from in vivo animal experiments were examined for the expression of EphA2, p-EphA2 S897, Ki-67, and cleaved caspase-3 via immunohistochemical analyses as described previously^19^.

### Cell proliferation, viability, wound healing, and invasion assays

Cell proliferation and viability were assayed using the MTT [3-(4,5-dimethylthiazol-2-yl)-2,5-diphenyltetrazolium bromide] method. Cell migration ability was assayed via the wound healing and/or Transwell approach, and the invasion ability of the cells was determined using a Matrigel-coated Transwell chamber device as described previously^21^.

### Drug screening

Small-scale HuH-7^R^-targeted quinazoline-derived small molecular compound screening was performed via IC_50_ ranking based on MTT assays. HuH-7^R^ cells were seeded into 96-well plates and exposed to concentration gradients of prazosin (Sigma-Aldrich), cyproheptadine (Sigma-Aldrich), phentolamine (Sigma-Aldrich), doxazosin (Sigma-Aldrich), terazosin (Sigma-Aldrich), WB-4101 (Sigma-Aldrich), tamsulosin (Sigma-Aldrich), clozapine (Sigma-Aldrich), bunazosin (Angene Chemical), and ketanserin (Sigma-Aldrich) for 72 h. The IC_50_ value of each compound was determined and ranked.

### Surface plasmon resonance (SPR) analysis

Further examination of the binding affinity (*K*_D_) between prazosin and EphA2 was conducted via SPR analysis with a Biacore T-200 system (GE Healthcare). Briefly, human recombinant EphA2 (R&D) as the ligand was coated on the CM5 sensor chip, and an analyte gradient of prazosin from 0 to 25 μM was assayed in running buffer containing 0.05% Tween-20 and 1% dimethyl sulfoxide in phosphate-buffered saline at a flow rate of 10 μL/min with a contact time of 60 s.

### Molecular dynamic (MD) simulation

The interactions between prazosin and EphA2 were further evaluated via MD simulations with the AMBER 16 software^22^. The crystal structures of human EphA2 LBD (PDB entry: 3CZU) and prazosin (the ligand from PDB entry: 3OWX) adopted for docking and MD simulations were obtained from the RSCB Protein Databank^23–25^. Briefly, the protonated coordinate file was prepared with the PDB2PQR tool with pH set as 7^26^. The prazosin docking model was established using Autodock Vina to generate the starting configurations for the subsequent MD simulations^27^. Energy minimization was conducted before the MD simulations, in which the Particle Mesh Ewald method was used for the treatment of long-range Coulomb interactions^28,29^. The Berendsen weak-coupling method was used for temperature regulation in the MD simulations, with the temperature set at 293 K and the coupling time constant at 0.1 ps^30^. The production run was set as indicated above with a non-bond cut-off of 10 Å, and a total run time of 100 ns. Hydrogen bonds were calculated with the CPPTRAJ program (cut-off: 3.5 Å distance, 120° angle). The molecular graphics of prazosin and EphA2 were generated using UCSF Chimera^31^.

### Analysis of synergistic activity

To analyze the combinatorial effect of EphA2 inhibition by sorafenib on HuH-7^R^ cells, the combination indices (CIs) of prazosin and sorafenib were evaluated as described previously with the CompuSyn software based on the Chou–Talalay theory^32^. CI < 1, CI = 1, and CI > 1 represent synergistic, additive, and antagonistic effects, respectively.

### Subcutaneous xenograft tumor models

Mouse subcutaneous tumor xenografts were established as described in a previous report^15^. After the tumor volumes reached ~200 mm^3^, the mice were divided into two groups: the tumor growth monitoring and prolonged survival evaluation groups. Each group was randomized into 4 subgroups and administered the following treatments: vehicle, 30 mg/kg/day sorafenib, 3 mg/kg/day prazosin, or both treatments. For tumor growth monitoring, tumor dimensions and volumes were measured and recorded, after which the mice were euthanized^15^. The endpoint for prolonged survival was set as either death or a tumor volume reaching 2000 mm^3^, following which the mice were euthanized^33^. Prolonged survival was estimated using the Kaplan–Meier method^33^. The tumor burden of all the mice in the two independent sets of experiments followed the restriction of an average tumor diameter of no more than 20 mm (volume < 4000 mm^3^). All animal studies followed the guidelines of the Institutional Laboratory Animal Care and Use Committee of National Taiwan University.

### Statistical analysis

Cell proliferation, viability, migration, invasion, and apoptosis assays were performed at least three times. Representative results are shown. Paired *t* test was used for the analysis of significant differences between groups. The level of statistical significance was set at *p < 0.05, **p < 0.01 or ***p < 0.001. The values for all measurements are expressed as the means ± SDs or SEMs.

## Results

### Identification, quantification, and bioinformatic analyses of differentially expressed phosphoproteins in HuH-7 and HuH-7^R^ cells

**Fig. 1:**
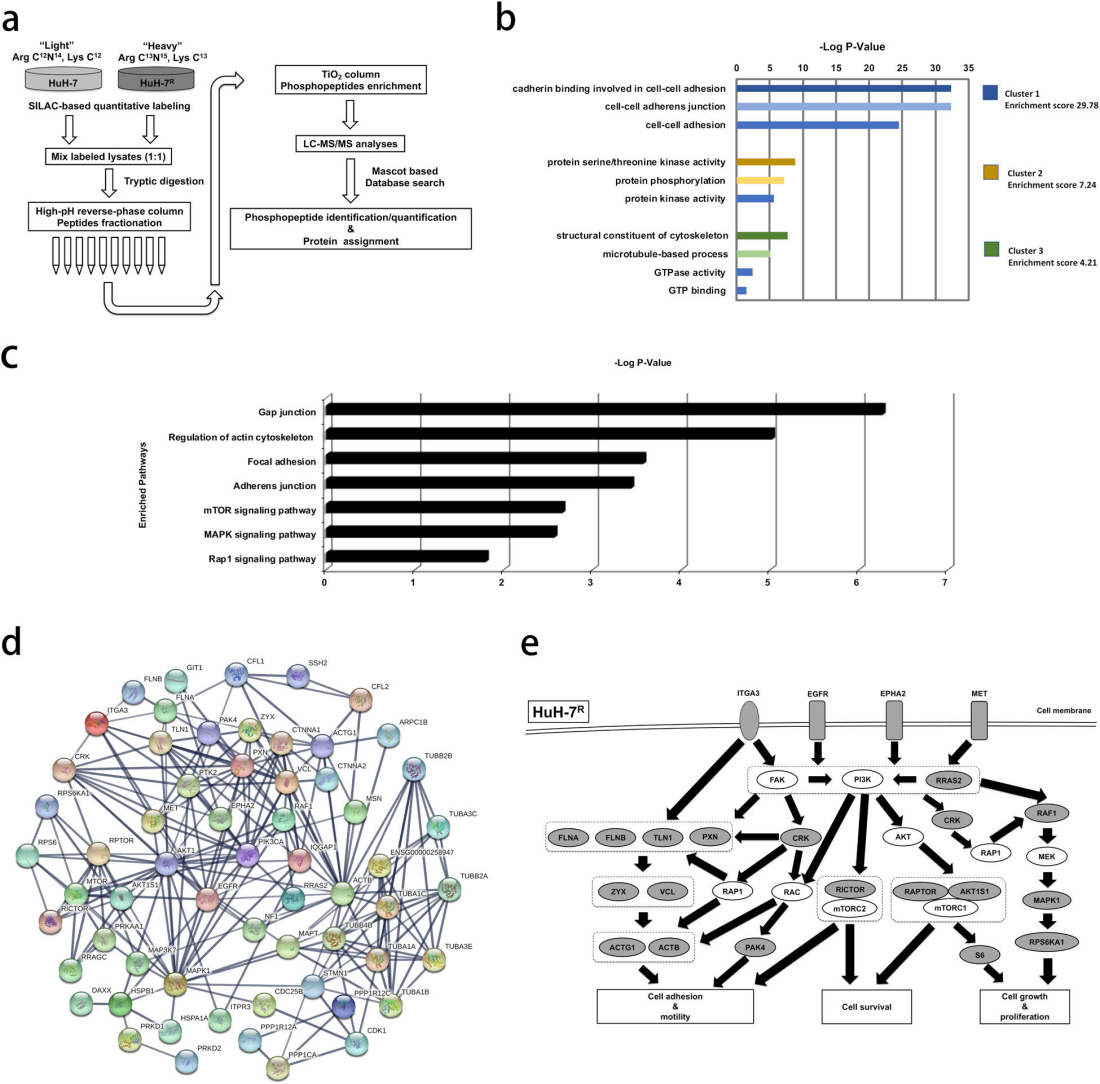
Differential phosphoproteomics between parental (HuH-7) cells and cells with acquired resistance to sorafenib (HuH-7^R^). **a** Workflow for quantitative phosphoproteomic analyses between parental (HuH-7) and HCC cells with acquired sorafenib resistance (HuH-7^R^) via SILAC-based mass spectrometry. Heavy and light cell lysates were mixed and digested with trypsin and fractionated by high-pH reverse-phase chromatography. Phosphopeptides were then purified with TiO_2_ column and analyzed in an LTQ-Orbitrap Velos hybrid mass spectrometer. **b** Clustered gene ontology functional enrichment was assessed with DAVID. Upregulated phosphoproteins in HuH-7^R^ cells showing a SILAC fold change H/L ≥ 1.5 were analyzed. The top three functional clusters are listed. −Log (*p* values) and enrichment scores are presented. **c** Pathway enrichment analysis of the upregulated phosphoproteins in HuH-7^R^ cells based on the KEGG pathway database with DAVID. Pathways with *p* values < 0.05 are shown. **d** Interaction linkage analysis of the molecules in enriched pathways shown in **c** with STRING. The connected molecules are shown. **e** Schematic representation of the postulated dysregulated phosphoprotein functional network in sorafenib-resistant HCC (HuH-7^R^) cells. Gray, identified upregulated phosphoproteins; white, molecules that are postulated but not defined; black bold arrows, signaling linkages.

To clarify the mechanistic pathway changes related to sorafenib resistance development in HuH-7^R^ cells relative to parental HuH-7 cells, we combined SILAC quantitative labeling with a phosphoproteomic platform. Resistant HuH-7^R^ cell lines were established previously^15^. The schematic design of the quantitative phosphoproteomic experiment for elucidating functional pathway alterations associated with the acquisition of sorafenib resistance between HuH-7 and HuH-7^R^ cells is shown in Fig. 1a. SILAC-labeled lysates were digested with trypsin and fractionated by high-pH reverse-phase chromatography, after which the TiO_2_-enriched phosphopeptides were analyzed in a Thermo LTQ Orbitrap Velos mass spectrometer. Subsequent identification and quantification were conducted using Thermo Proteome Discoverer with the Mascot algorithm. The dataset was filtered according to a FDR < 1% via target-decoy analysis for reliability^34^. In the three independent replicate experiments, SILAC-based phosphoproteomic analyses yielded an average of approximately 5000 quantified phosphopeptides corresponding to an average of approximately 1500 phosphoproteins. As typically observed, the majority of phosphopeptides identified were phosphorylated at serine or threonine, consistent with the results of phosphorylation profiling with pan P-Ser, P-Thr, and P-Tyr antibodies (data not shown). Owing to the considerable hyperphosphorylated protein enrichment, our subsequent focus was on these upregulated phosphoproteins. Prior to bioinformatic analyses, a threshold of >1.5-fold was considered to indicate a significant increase in phosphorylated proteins in resistant HuH-7^R^ cells, which included 533 phosphoproteins. To gain insight into the sorafenib resistance-associated functional alterations in the upregulated phosphoproteome, we first subjected the 533 upregulated phosphoproteins to clustered gene ontology functional enrichment analysis based on the molecular function, cellular compartment, and biological process categories with DAVID. The top 3 functional clusters that were enriched (among which the subcategories exhibited *p* values < 0.05) are shown in Fig. 1b and Table 1. The top enriched functions included cell adhesion, junction-related functions, protein phosphorylation, kinase-related functions, and functions associated with cytoskeleton modulation. Notably, two of the top enriched clusters showed that the significantly altered functions integrated from the upregulated phosphoproteome in sorafenib-resistant HCC cells were related to the cancer hallmarks of invasion and metastasis.

**Table 1:** Top 3 functional clusters enriched from the upregulated (fold change >1.5) phosphoproteins in HuH-7^R^ cells.

		Count	*p* Value
*Annotation cluster 1*	*Enrichment Score: 29.78*		
GOTERM_MF_DIRECT	Cadherin binding involved in cell-cell adhesion	60	3.80E−33
GOTERM_CC_DIRECT	Cell–cell adherens junction	62	3.90E−33
GOTERM_BP_DIRECT	Cell–cell adhesion	50	3.10E−25
*Annotation cluster 2*	*Enrichment Score: 7.24*		
GOTERM_MF_DIRECT	Protein serine/threonine kinase activity	36	1.40E−09
GOTERM_BP_DIRECT	Protein phosphorylation	37	5.90E−08
GOTERM_MF_DIRECT	Protein kinase activity	29	2.30E−06
*Annotation cluster 3*	*Enrichment Score: 4.21*		
GOTERM_MF_DIRECT	Structural constituent of cytoskeleton	18	1.80E−08
GOTERM_BP_DIRECT	Microtubule-based process	9	7.10E−06
GOTERM_MF_DIRECT	GTPase activity	16	3.60E−03
GOTERM_MF_DIRECT	GTP binding	19	3.20E−02

*MF* molecular function, *CC* cellular function, *BP* biological process.

### Pathway enrichment and linkage interaction network of upregulated phosphoproteins

To further elucidate the potential pathways through which these upregulated phosphoproteins are involved in the regulation of the disrupted functions associated with sorafenib resistance indicated above, all 533 upregulated phosphoproteins were additionally subjected to pathway enrichment analysis based on the KEGG pathway database with DAVID. The significantly (*p* value < 0.05) canonically enriched pathways are shown in Fig. 1c and Table 2. The data showed that the major altered pathways in HCC cells with acquired sorafenib resistance included the gap junction, regulation of cytoskeleton, focal adhesion, adherens junction, mammalian target of rapamycin (mTOR) signaling, MAPK signaling, and Rap1 signaling pathways. To understand the interacting networks and altered pathways, all the non-redundant molecules in the seven significantly enriched pathways indicated in Fig. 1c and Table 2 were subjected to STRING analysis. Interestingly, we observed the formation of an interconnected functional network involving most of the analyzed molecules, which may also present functional linkage with the PI3K/Akt pathway (Fig. 1d). To summarize the major pathway alterations in sorafenib-resistant HCC cells, we further inferred a postulated mechanistic scheme from the results presented in Fig. 1d along with reference searches (Fig. 1e). Interestingly, the upregulated phosphoproteins were involved in dysregulated pathways such as the focal adhesion kinase (FAK), PI3K/Akt, mTOR, and Ras-Raf-MAPK pathways and further transduced the effects on cellular functions, such as cell adhesion, motility, survival, growth and proliferation. Among the altered functional networks, four upstream membrane receptors, EphA2, EGFR, MET (hepatocyte growth factor receptor), and ITGA3 (integrin alpha-3), were further identified. By integrating phosphoproteomic data and bioinformatic analyses, an altered functional network associated with the functions of cell motility, survival, growth, and proliferation that may aid in the development of sorafenib resistance in HCC cells was revealed.

**Table 2:** Enriched pathways of the upregulated phosphoproteins (fold change >1.5) in HuH-7^R^ cells by DAVID based on KEGG pathways.

Enriched pathway	−Log (*p* value)	Molecules
Gap junction	6.262012674	RAF1, CDK1, **EGFR**, ITPR3, MAPK1, MAP3K2, TUBA1A, TUBA1B, TUBA1C, TUBA3C, TUBA3E, TUBB2A, TUBB2B, TUBB3, TUBB4B
Regulation of actin cytoskeleton	5.018181393	CRK, GIT1, IQGAP1, RAF1, ACTB, ACTG1, ARPC1B, CFL1, CFL2, **EGFR**, **ITGA3**, MAPK1, MSN, PAK4, PXN, PPP1CA, PPP1R12A, PPP1R12C, RRAS2, SSH2, VCL
Focal adhesion	3.568636236	CRK, **MET**, RAF1, ACTB, ACTG1, **EGFR**, FLNA, FLNB, **ITGA3**, MAPK1, PAK4, PXN, PPP1CA, PPP1R12A, PPP1R12C, TLN1, VCL, ZYX
Adherens junction	3.432973634	IQGAP1, **MET**, ACTB, ACTG1, CTNNA1, CTNNA2, **EGFR**, MAPK1, MAP3K7, VCL
mTOR signaling pathway	2.657577319	AKT1S1, RICTOR, RRAGC, MAPK1, PRKAA1, RPTOR, RPS6KA1, RPS6
MAPK signaling pathway	2.568636236	CRK, RAF1, CDC25B, DAXX, **EGFR**, FLNA, FLNB, HSPA1A, HSPB1, MAPT, MAPK1, MAP3K2, MAP3K7, NF1, RRAS2, RPS6KA1, STMN1, ZAK
Rap1 signaling pathway	1.795880017	CRK, **EPHA2**, **MET**, RAP1GAP, RAF1, RAPGEF6, ACTB, ACTG1, **EGFR**, MAPK1, PRKD1, PRKD2, SIPA1, TLN1

Membrane receptors are specifically marked in bold.

### EphA2 is a novel aberrantly activated membrane receptor in the altered functional network

**Fig. 2:**
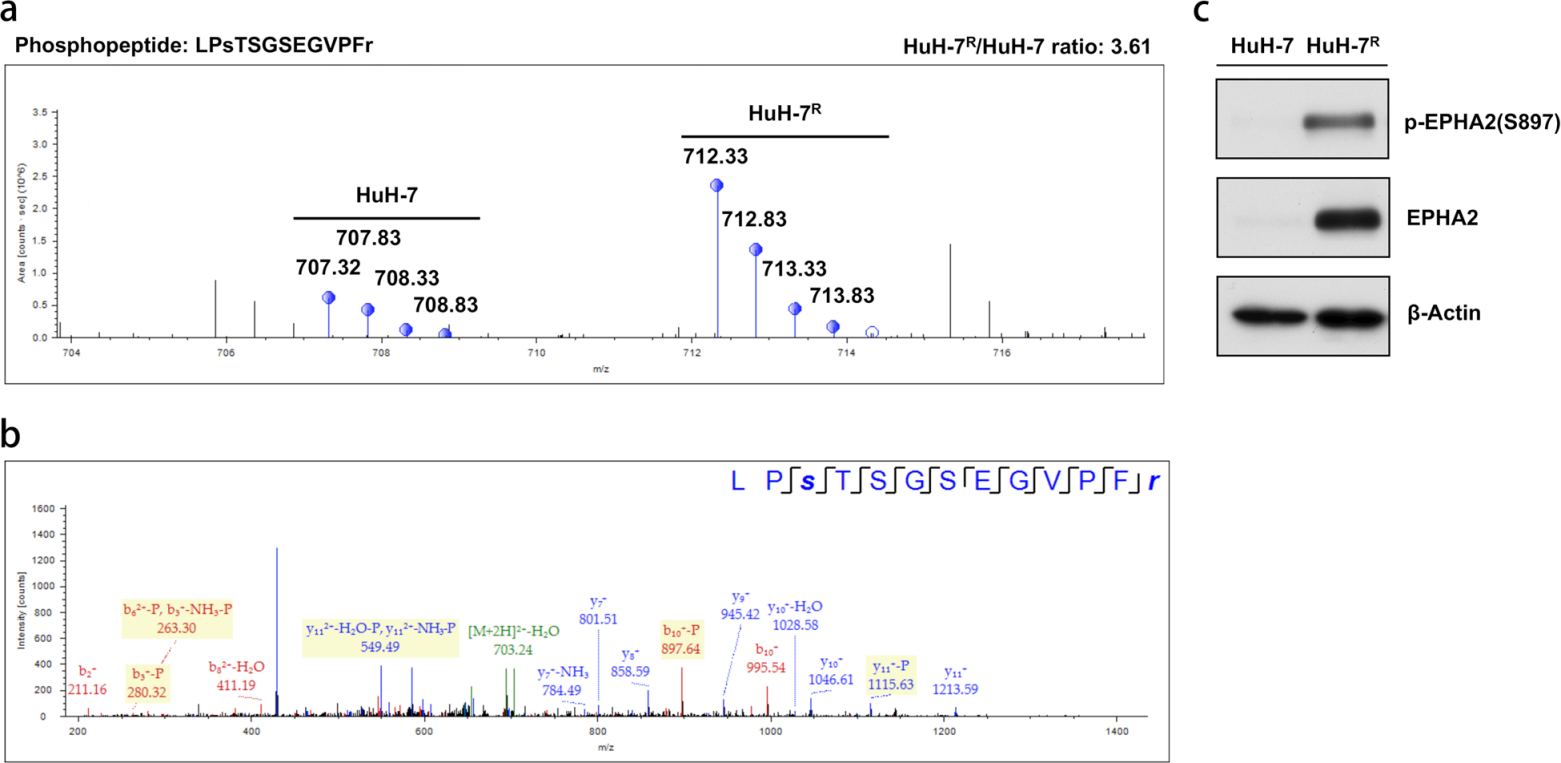
The potential novel RTK candidate EphA2 for the modulation of drug resistance identified via quantitative MS. **a** The quantified MS spectra of the identified SILAC-labeled phosphopeptide of EphA2 are shown. **b** MS/MS spectra of the corresponding identified phosphopeptide of EphA2. **c** Validation of the change in the identified phosphorylation site of EphA2 between HuH-7 and HuH-7^R^ cells via western blotting. The identified peptide sequence, phosphorylated residue, and quantified ratio are presented. EPHA2 ephrin type A receptor 2.

Given the results of the functional and pathway analyses, a functional network primarily associated with cell motility linked by potentially altered downstream pathways was revealed. The four upstream receptors were first observed within this dysregulated network. Owing to the prominent connection of the aberrant functions with cell motility, we next focused on potential targets closely related to this function. Accordingly, a newly identified RTK, EphA2, caught our attention among the altered pathways; EphA2 is intimately related to the regulation of cell migration and adhesion associated with both physiological and pathological status. The SILAC-based quantitative MS and MS/MS spectra of the characteristic phosphopeptide of EphA2 are shown in Fig. 2a, b. The aberrantly activated phosphorylation site of EphA2 was observed to be Ser897. Next, the phosphorylation status of Ser897 of EphA2 in HuH-7^R^ cells was further validated via western blotting. The data from western blot analyses were consistent with those of phosphoproteomic analyses, and both analyses showed that Ser897 of EphA2 is highly phosphorylated in HuH-7^R^ cells compared to parent HuH-7 cells (Fig. 2c). To further examine the EphA2 expression levels among other HCC cell lines, we collected three other HCC cell lines, PLC-5, Sk-Hep-1 and Hep3B, and determined the expression level of EphA2 and S897 phosphorylation status by western blotting (Supplementary Fig. 1a). Then we compared the sorafenib sensitivity (IC_50_) of these cell lines (Supplementary Fig. 1b). We found that Sk-Hep-1 cells expressed high levels of EphA2 and p-EphA2 S897 and presented a high sorafenib IC_50_, which was slightly higher than that of HuH-7^R^ cells. Furthermore, several studies have reported that, under Akt activation, EphA2 phosphorylation at Ser897, which has been reported as a potential site regulated by Akt, drives tumor cells toward oncogenic survival and metastatic pathways^35^. Taking into consideration the aberrantly upregulated phosphorylation of the oncogenic Ser897 of EphA2 and the high relevance of EphA2 function to the observed alteration of cell motility in resistant cells, we further investigated the role of EphA2 and its potential correlation with sorafenib resistance in HuH-7^R^ and Sk-Hep-1 cells.

### EphA2 knockdown inhibits proliferation, tumorigenicity, migration, and invasion and promotes sorafenib sensitivity in HCC cell lines

**Fig. 3:**
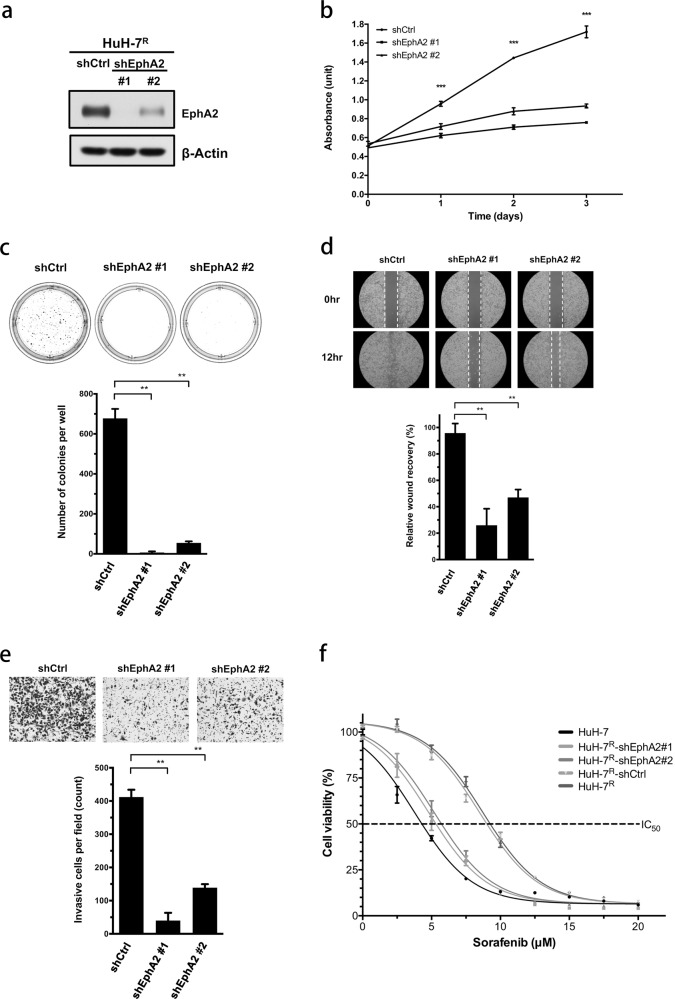
EphA2 mediates proliferation, migration, invasion, and sorafenib sensitivity. **a** HuH-7^R^ cells were infected with lentiviruses containing shEphA2 (#1, #2) or control shRNA (shCtrl) and, 48 h later, were lysed and analyzed by western blotting with the indicated antibodies. **b** The viability of EphA2 knockdown HuH-7^R^ cells was determined at the indicated time points with the MTT assay. The plots depict cumulative cell numbers versus days in culture. **c** The tumorigenicity of EphA2 knockdown HuH-7^R^ cells was determined using the soft agar colony-formation assay. **d** Wound-healing assay of shEphA2-infected HuH-7^R^ cells. The micrographs show cells that migrated into the gap 0 and 24 h after the removal of the insert. **e** Transwell invasion assay of shEphA2-infected HuH-7^R^ cells. Cells in the central field of each insert were visualized via light microscopy and quantified. **f** HuH-7, shCtrl, and shEphA2-containing HuH-7^R^ cells were exposed to sorafenib at the indicated concentrations for 72 h, and cell viability was analyzed with the MTT assay. The concentration–response curve for sorafenib in the EphA2 knockdown group shifted toward a lower concentration compared to that for shCtrl-infected HuH-7^R^ cells. All statistical data were calculated from three independent replicates (**p < 0.01; ***p < 0.001; shCtrl control shRNA, shEphA2 shRNA against EphA2).

Following the knockdown of EphA2 expression, several functional assays were performed to clarify the potential role of EphA2 in HCC cell lines. The lentiviral-mediated delivery of EphA2 shRNA was employed to suppress EphA2 expression in HuH-7^R^ and Sk-Hep-1 cells. The knockdown efficiency is shown in Fig. 3a and Supplementary Fig. 2a. In the MTT assay, the proliferation of HuH-7^R^ and Sk-Hep-1 cells was significantly suppressed after EphA2 knockdown (Fig. 3b and Supplementary Fig. 2b). The anchorage-independent colony-forming ability (tumorigenicity) was additionally impaired after the inhibition of EphA2 expression in HuH-7^R^ cells (Fig. 3c). Wound healing (Fig. 3d and Supplementary Fig. 2c) and Transwell (Fig. 3e and Supplementary Fig. 2d) assays showed decreased motility of HuH-7^R^ and Sk-Hep-1 cells following the suppression of EphA2 compared to the control groups of cells. The collective results indicated that EphA2 affects several aspects of cancer biology in HuH-7^R^ and Sk-Hep-1 cells. Our next key concern was the relevance of EphA2 in sorafenib drug resistance. To address this issue, the drug sensitivity of EphA2-depleted HuH-7^R^ and Sk-Hep-1 cells was compared with that of parental control cells. As indicated in Fig. 3f, the IC_50_ values of the two EphA2-suppressed HuH-7^R^ cell lines were shifted toward that of HuH-7 cells, while the IC_50_ values of mock and control HuH-7^R^ cells were twice that of HuH-7 cells. The same increase in sorafenib sensitivity was observed in Sk-Hep-1 cells after EphA2 suppression (Supplementary Fig. 2e). In summary, knockdown-based functional assays demonstrated that EphA2 not only modulates growth and progression but also influences sorafenib sensitivity in both HCC cell lines.

### EphA2 S897 phosphorylation is important for several aspects of EphA2 oncogenic activity in HuH-7^R^ cells

To validate the importance of the S897 phosphorylation of EphA2, we performed mutational assays in HuH-7^R^ cells. An EphA2 S897A mutant was constructed in a lentivirus expression-based vector. The overexpression of control wild-type EphA2 and the S897A mutant in HuH-7^R^ cells was accomplished via the lentivirus-mediated delivery method. The expression efficiency is shown in Supplementary Fig. 3a. In the MTT assay, the proliferation of HuH-7^R^ cells was significantly suppressed after the abolishment of EphA2 S897 phosphorylation (Supplementary Fig. 3b). Wound healing (Supplementary Fig. 3c) and Transwell (Supplementary Fig. 3d) assays showed decreased motility of HuH-7^R^ cells following the inhibition of EphA2 S897 phosphorylation compared to the control group of cells. Furthermore, the drug sensitivity of the EphA2 S987A mutant and wild-type HuH-7^R^ cells was also compared. As indicated in Supplementary Fig. 3e, the IC_50_ values of the EphA2 mutant HuH-7^R^ cell lines were shifted toward lower IC_50_ values compared with those of mock and EphA2 wild-type HuH-7^R^ cells. The collective results indicate that S897 phosphorylation status affects several aspects of the oncogenic activity of EphA2 in HuH-7^R^ cells.

### Targeting the ligand-binding domain (LBD) of EphA2 may suppress Akt activity in HuH-7^R^ cells

**Fig. 4:**
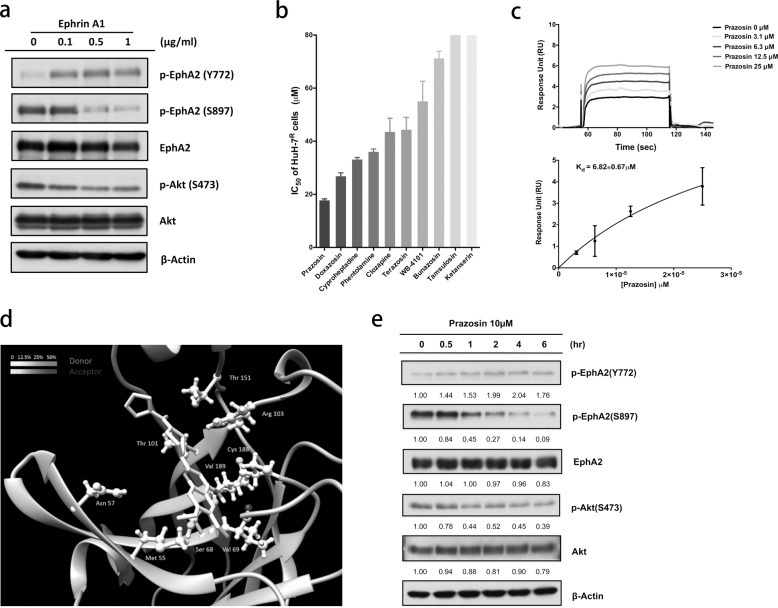
Screening of small molecular agonists for EphA2-targeted inhibition. **a** Ligand-dependent inhibition of oncogenic activity of EphA2 and Akt phosphorylation in HuH-7^R^ cells. HuH-7^R^ cells were treated with Ephrin-A1-Fc at the indicated concentrations. Cell lysates were separated via SDS-PAGE and analyzed using western blotting with the specified antibodies. **b** The small-scale screening of quinazoline-based compounds was performed and the IC_50_ values for HuH-7^R^ cells were determined with the MTT assay. **c** Validation of the EphA2-binding affinity of prazosin analyzed via the surface plasmon resonance (SPR) assay. Representative binding response and saturation curves are shown. **d** The molecular dynamic simulation of prazosin complexed with EphA2 was performed with the AMBER 16 software using the crystal structures of EphA2 (3CZU) and prazosin (3OWX) adopted from the RSCB Protein Databank. The molecular plot was generated using UCSF Chimera. The presented amino acids are potentially involved in hydrogen bonding interactions with prazosin, with the level of likelihood displayed in shades of gray. **e** Inhibitory effects of prazosin on EphA2 and Akt phosphorylation in HuH7^R^ cells. HuH7^R^ cells were treated with 10 μM prazosin for the indicated times. The cell lysates were separated via SDS-PAGE and analyzed using western blotting with the specified antibodies. The intensity was quantified via densitometry and normalized to that of β-actin.

EphA2 plays exclusive roles in cancer progression^36^. The Ser897 phosphorylation of EphA2 by Akt is proposed to participate in tumor cell migration, invasion, and drug resistance^37^. The modulation of EphA2 signaling may therefore represent a novel strategy for reversing drug resistance and inhibiting malignant cancer progression. Recent studies have revealed that quinazoline-based compounds display antineoplastic activity^38^. To examine this hypothesis in HuH-7^R^ cells, we first used Ephrin A1 (Ephrin-A1-Fc), the natural ligand of EphA2, to investigate the inhibitory effect on EphA2 and Akt activity (Fig. 4a). The treatment of HuH-7^R^ cells with the Ephrin A1 ligand induced an increase in the phosphorylation of EphA2 Tyr772, further suppressing EphA2 Ser897 phosphorylation and leading to a decrease in Akt Ser473 phosphorylation. Next, we performed small-scale quinazoline-based derivative screening from available sources to identify EphA2-targeting small molecules (Fig. 4b). In the cell viability assay, HuH-7^R^ cells showed the highest sensitivity to prazosin. Next, we ascertained whether prazosin inhibits HuH-7^R^ cells by targeting EphA2. SPR analysis method adopted for EphA2-binding evaluation showed that prazosin interacts with the EphA2 extracellular domain (ECD) with an affinity (*K*_D_) of ~7 μM (Fig. 4c). To confirm the binding site of prazosin in the EphA2 ECD, we applied a MD simulation to compute the hydrogen bond interactions between the EphA2 LBD (the major binding site in the ECD) and prazosin (Fig. 4d). Prazosin formed hydrogen bonds with specific residues of the EphA2 LBDs, such as Asn 57, Ser 68, Val 69, and Arg 103 (Supplementary Fig. 4a). We further investigated the effects of the small-molecule targeting of EphA2 LBD on Akt activity in the HuH-7^R^ and Sk-Hep-1 cell lines. The targeting of the EphA2 LBD by prazosin with the upregulation of Tyr772 phosphorylation led to the inhibition of Akt with the suppression of Ser473 phosphorylation (Fig. 4e and Supplementary Fig. 4c). The characteristic upregulation of Ser897 phosphorylation was also decreased, suggesting that prazosin interacts with the EphA2 LBD and inhibits Akt kinase activity in an EphA2-dependent manner.

### The regulation of EphA2 with a ligand mimic induces apoptosis and suppresses motility in HuH-7^R^ cells

**Fig. 5:**
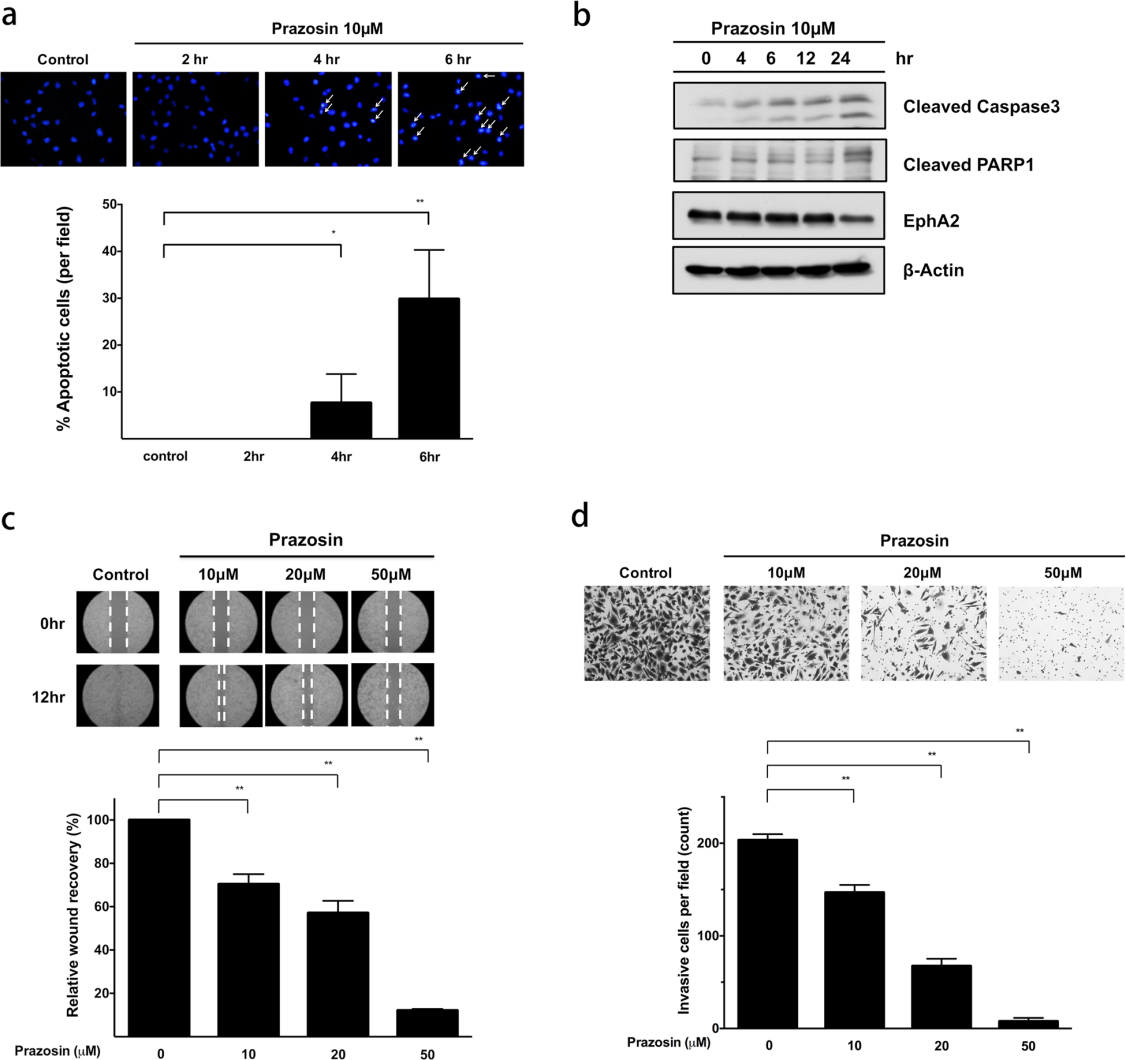
Prazosin induces cell apoptosis and suppresses metastasis. **a** Apoptosis induced by prazosin was evaluated using Hoechst 33342 staining. HuH-7^R^ cells were treated with 10 μM prazosin at different time points, and the number of apoptotic cells was quantified. The white arrows indicate cells with fragmented nuclei. **b** Prazosin exerts an apoptotic effect on HuH-7^R^ cells. Cells were treated with prazosin at the indicated concentrations for 48 h. Cell lysates were separated via SDS-PAGE and analyzed by western blotting using the indicated antibodies. **c** Evaluation of the inhibitory effect of the indicated concentrations of prazosin on HuH-7^R^ cell migration with the wound-healing assay. Micrographs show cells that migrated into the gap at 0 and 12 h. **d** Inhibition of HuH-7^R^ cell invasion by prazosin at the indicated concentrations. Cells in the central field of each insert were visualized via light microscopy and quantified. All statistical data were calculated from three independent replicates (*p < 0.05; **p < 0.01).

The ligand activation of EphA2 is reported to trigger receptor internalization and lead to cell rounding and subsequent death^39^. Accordingly, we investigated whether the EphA2 agonist activity of prazosin is specific to resistant cellular function. Immunofluorescent membrane staining and confocal microscopy assays showed that prazosin treatment significantly abolished the membrane localization of EphA2 and triggered its internalization in a time-course experiment (Supplementary Fig. 4b). Following internalization, we evaluated the effects on cell death. Specifically, Hoechst 33342-based apoptosis assays were performed in cells under prazosin treatment. The status of nuclear DNA fragmentation and pro-apoptotic enzyme (caspase-3 and PARP) activity in HuH-7^R^ cells were monitored (Fig. 5a, b). Our data indicate that the ligand mimic targeting of EphA2 by prazosin inhibits EphA2 and induces cellular apoptosis. As specified above, the Akt activation of EphA2 increases tumor cell motility by promoting migration and invasion. Accordingly, we examined the influence of prazosin, targeting EphA2, on HuH-7^R^ cell migration and invasion (Fig. 5c, d). HuH-7^R^ cell motility was suppressed in a dose-dependent manner in the prazosin treatment group. In summary, the ligand-dependent modulation of EphA2, for example, by the prazosin agonist, not only facilitates the blockage of malignancy development but also induces the apoptosis of drug-resistant cells.

### Combinatorial prazosin treatment enhances sensitivity to sorafenib in HuH-7^R^ cells

**Fig. 6:**
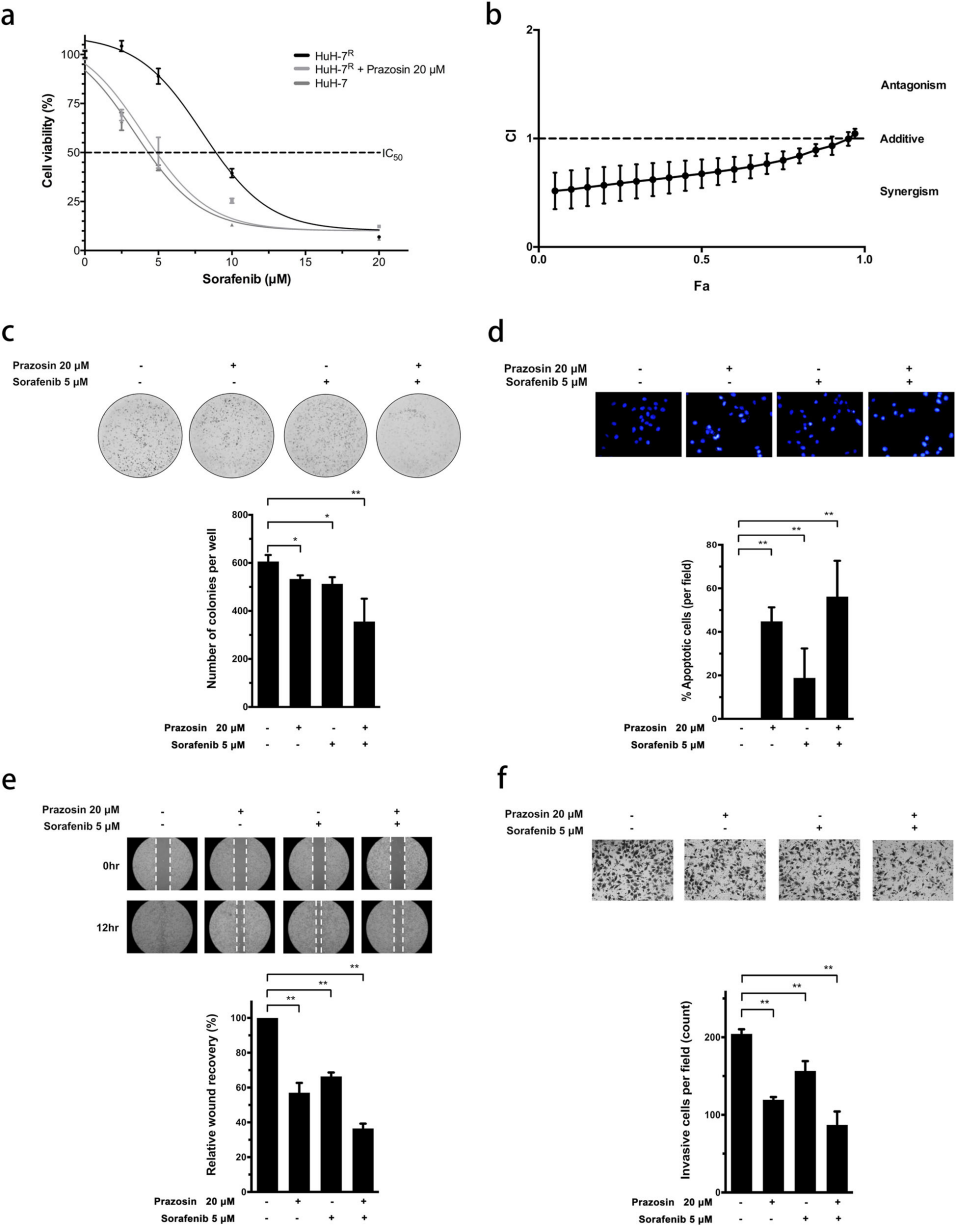
Prazosin shows synergistic activity with sorafenib to inhibit cell growth, tumorigenicity, migration, and invasion in HuH7R cells. **a** HuH-7 and HuH-7^R^ cells were exposed to varying concentrations of sorafenib either alone or in combination with 20 μM prazosin for 72 h, and viability was measured via the MTT assay. **b** The combined effects of prazosin (20 μM) and sorafenib (5 μM) in HuH-7^R^ cells were measured via the MTT assay. Combination index (CI) plots were generated using CompuSyn (CI < 1, synergistic; CI = 1, additive; CI > 1, antagonistic). Fa effect under each concentration, CI combination index. **c** Combined effect of prazosin and sorafenib on the tumorigenic ability of HuH-7^R^ cells. HuH-7^R^ cells were treated with prazosin and sorafenib at the indicated concentrations for 14 days, and the colony-formation assay was performed. **d** Synergistic effect of prazosin and sorafenib on apoptosis in resistant cancer cells. HuH-7^R^ cells were treated with prazosin and sorafenib at the indicated concentrations, followed by staining with Hoechst 33342, and the number of apoptotic cells was quantified. **e** Effect of the combination of prazosin and sorafenib on wound healing in HuH-7^R^ cells. The micrographs show cells that had migrated into the gap 0 and 12 h after the removal of the insert. **f** Synergistic effect of prazosin and sorafenib on the invasion of HuH-7^R^ cells examined via the Transwell assay. Cells in the central field of each insert were visualized via light microscopy and quantified. All statistical data were calculated from three independent replicates (*p < 0.05; **p < 0.01).

Given the anti-oncogenic effects of the ligand mimic modulation of EphA2, we further examined the influence of prazosin on sorafenib sensitivity in HuH-7^R^ cells. The comparison of the IC_50_ values for sorafenib between control and prazosin-targeted HuH-7^R^ cells revealed that the simultaneous modulation of EphA2 restores sorafenib sensitivity toward the level observed in parental HuH-7 cells (Fig. 6a). In addition, the concomitant suppression of EphA2 with prazosin and sorafenib had a synergistic inhibitory effect on resistance (Fig. 6b). The anchorage-independent colony-formation assay revealed that tumorigenicity was more significantly inhibited in HuH-7^R^ cells subjected to combined treatment relative to treatment with the single agents (Fig. 6c). Moreover, in the apoptosis assay (Fig. 6d), sorafenib treatment coupled with EphA2 suppression induced cell death to a more significant extent in HuH-7^R^ cells. The combination of EphA2 targeting with sorafenib treatment led to increased suppression of cell movement and motility in HuH-7^R^ cells (Fig. 6e, f). Thus the simultaneous targeting of EphA2 along with sorafenib treatment may represent a potential therapeutic strategy for Akt-overactivated HCC with drug resistance.

### Prazosin augments the activity of sorafenib in vivo

**Fig. 7:**
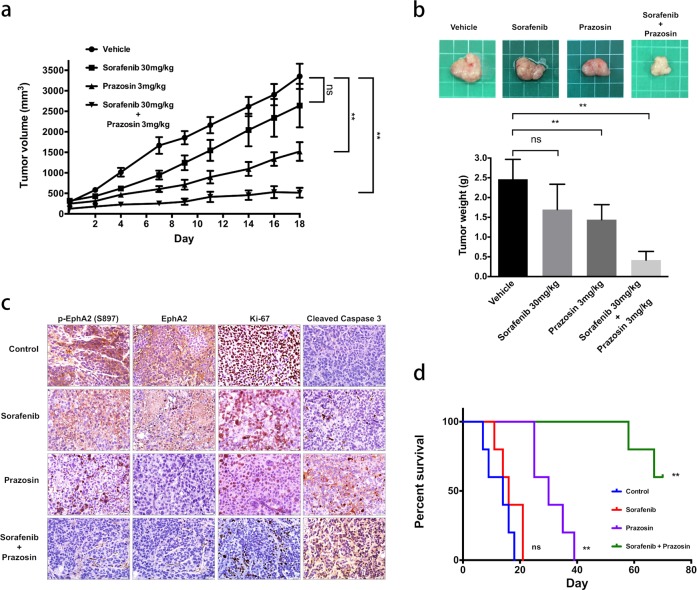
Prazosin overcomes sorafenib resistance in vivo. HuH-7^R^ cells were subcutaneously injected into nude mice. At a tumor volume of ~200 cm^3^, mice were orally treated with vehicle (*N* = 5), sorafenib at 30 mg/kg (*N* = 5), and/or prazosin at 3 mg/kg (*N* = 5) for 18 days. **a** Measurement of tumor volumes. **b** Upper, representative images of tumor xenografts. Lower, tumor weights after the end point. **c** Tumor sections were analyzed via immunohistochemistry. Paraffin-embedded sections of tumor tissue were stained with the indicated antibodies. Representative sections at ×400 magnification. **d** Survival times were calculated with the Kaplan–Meier method. Statistical data were calculated from five replicates under a single independent experimental design (ns not significant; **p < 0.01).

To further assess the efficacy of the combined treatment against sorafenib-resistant HCC in vivo, we employed mouse xenograft tumor models generated via the subcutaneous injection of HuH-7^R^ cells. As indicated in Fig. 7a, HuH-7^R^ cells treated with sorafenib alone showed marginal inhibition of tumor growth that was not statistically significant. In contrast, tumors treated with prazosin alone or the combined regimen were significantly suppressed. In addition, the tumor burden was markedly decreased in the group subjected to prazosin or combined treatment compared with the control and sorafenib treatment groups (Fig. 7b). The immunohistochemical assessment of key marker proteins in the tumor sections of each group revealed a significant reduction in EphA2 expression and activity in the presence of prazosin (Fig. 7c). Tumor proliferation (Ki-67) and apoptosis (cleaved caspase-3) were markedly altered under the combination of EphA2 targeting with sorafenib treatment (Fig. 7c). Kaplan–Meier analysis of tumor growth rates predicted significantly prolonged survival in the combination therapy group compared with the other three groups (Fig. 7d). Taken together, our results clearly suggest that the combination of EphA2 targeting with sorafenib treatment represents a promising option for the reversal of drug resistance in HCC.

## Discussion

HCC involves complicated molecular aberrations and signaling pathways^40^. One of the key pathways involved in the growth and proliferation of HCC is MAPK signaling, which is frequently upregulated in cancer cells. Sorafenib is a multikinase inhibitor that is commonly used for the treatment of advanced HCC, but most patients eventually develop drug resistance^41^. Therefore, the elucidation of the molecular changes that underlie the biological pathways of acquired drug resistance and the discovery of new therapeutic targets are of critical importance for the development of effective HCC treatments.

In the present study, we applied quantitative phosphoproteomics to systematically investigate global phosphorylation changes between drug-resistant (HuH-7^R^) and parental (HuH-7) HCC cells in the context of the sorafenib response. Bioinformatic integration of the upregulated phosphoproteins revealed that certain alternatively dysregulated cellular pathways involved in cell adhesion, motility, cell survival, growth, and proliferation are implicated in promoting the development of acquired resistance in HCC cells. Among pathways, aberrations in cell adhesion and motility were the major changes in the functionality of resistant HCC cells. Interestingly, upstream of these dysregulated pathways, four receptors, EphA2, EGFR, MET, and ITGA3, were identified. First, the phosphorylation of S1042 of ITGA3 was indicated to alter the phosphorylation and function of FAK, paxillin, and P130^CAS^ and impacted cell adhesion and migration^42^. It has been previously reported that aberrant phosphorylation-activated MET levels contribute to sorafenib resistance in HCC, although the specific effect of the newly identified S1042 on MET and sorafenib sensitivity will need further clarification in the future^43^. Overactivated EGFR has been indicated to play a critical role in HCC sorafenib resistance^44^. While the detailed functional impacts of S1049/S1041 on EGFR observed here require further investigation, the activation of these sites has been noted under EGFR functional dysregulation^45^.

Further analyses of the downstream molecules revealed upregulated phosphoproteins belonging to the mTOR complex 1/2 (mTORC1/2) pathways, including AKT1S1, RPTOR, RICTOR, and RPS6. It was previously shown that the S202/S203 phosphorylation of AKT1S1 results in the activation of mTORC1 signaling^46^. RPTOR S863 phosphorylation activates and increases the activity of mTORC1^47^. RICTOR, a component of the mTORC2 complex, can affect the functions of the cytoskeleton, cell growth, and survival^48^. Therefore, the alteration of RICTOR phosphorylation may imply the existence of aberrant mTORC2 activity and subsequent cellular functions. In addition, the S235/S236 phosphorylation of RPS6 was revealed to be regulated by mTOR and subsequently promote translation and cell growth^49^. Here we observed significantly activated mTOR pathway-related molecules, which suggested possible highly upregulated upstream Akt activity, consistent with the comparison of the resistant (HuH-7^R^) and parental (HuH-7) cell lines via western blotting (data not shown). Apart from the mTOR pathways, the majority of the upregulated phosphoproteins were associated with functional pathways involved in cell motility and movement. A prominent pathway was FAK-modulated downstream signaling. PXN is an important molecule in the FAK–paxillin axis. The dysregulation of PXN phosphorylation and activity has been reported to impact focal adhesion and cell motility^50^. The S181 phosphorylation of PAK4 was suggested to alter its own activity and functions in cell adhesion and motility^51^. These findings suggested that the aberrant activity of PXN, PAK4, and other related downstream molecules indicated possible upstream RTK-FAK-mediated pathway activation and the promotion of metastatic ability.

More importantly, among this dysregulated functional network, we identified a novel potential upstream receptor, EphA2. A number of studies support important roles of EphA2 in cancer biology, including angiogenesis, tumorigenicity, proliferation, drug resistance, and particularly metastasis^37^. In our experiments, the inhibition of EphA2 function impaired tumor cell growth and motility and, most importantly, reversed the drug resistance of HuH-7^R^ cells. To examine the EphA2 expression levels in other HCC cell lines, we selected three other cell lines, PLC-5, Sk-Hep-1, and Hep3B. In our screens, Sk-Hep-1, which was one of two cell lines exhibiting a higher IC_50_ than HuH-7^R^ cells, showed strong expression of EphA2 with simultaneously upregulated S897 phosphorylation. In accord with the status of EphA2, Sk-Hep-1 cells also showed elevated Akt expression and active phosphorylation. In comparison, PLC-5 cells, which presented an IC_50_ slightly higher than that of HuH-7 cells, did not display increases in either EphA2 or Akt expression or activity. Tumor heterogeneity has been proposed and observed in many cancers, including liver cancer and lung cancer (non-small cell lung carcinoma)^52,53^. Drug resistance-induced tumor recurrence and treatment impediments have been attributed to heterogeneous tumor cell subpopulations, such as cancer stem cells^53^. Therefore, it is suggested that drug-resistant cancer cells, such as HuH-7^R^ and/or Sk-Hep-1 cells, may represent a certain subpopulation of a heterogeneous liver tumor. During sorafenib treatment, the resistant cell populations survived and/or developed drug resistance in patients who ultimately suffered recurrence and treatment failure.

EphA2 belongs to the ephrin receptors (EPHs), which are the largest family of RTKs. EPHs typically interact with their native ligands, ephrins, and display forward and reverse bidirectional signaling^54^. Based on earlier findings, EphA2 can play either a tumor-promoting or tumor-suppressive role, and it may adopt two different mechanisms to exert its effects on cancer biology, involving a ligand-dependent or ligand-independent pathway. In the ligand-dependent pathway, EphA2 is activated by EphrinA1 with subsequent tyrosine phosphorylation, while EphA2 is phosphorylated at S897, which is reported to be mediated by Akt in a ligand-independent manner^37^. Thus the phosphorylation of EphA2 at S897 in resistant HCC cells should promote ligand-independent signaling and drive tumor-promoting activity. Similarly, in BRAF inhibitor-treated melanoma, the activation of EphA2 ligand-independent signaling is reported to be responsible for the promotion of metastasis and treatment failure^55^. In agreement with our mutational assays, impaired S897 phosphorylation could compromise the tumor-promoting activities of EphA2 in cell growth, migration, invasion and, most importantly, sorafenib sensitivity in resistant cells (HuH-7^R^) relative to control cells overexpressing wild-type EphA2.

However, the activation of the ligand-dependent functionality of EphA2 by the native ligand Ephrin A1 would lead to receptor internalization, degradation, and subsequent EphA2-dependent Akt activity inhibition^56^. Moreover, ligand mimic small molecular or peptide agonists that target the EphA2 LBD, activate EphA2 ligand-dependent signaling, and inhibit cancer cell migration and metastasis^57^. In accord with our results obtained via prazosin treatment, an earlier study showed that a quinazoline-like small molecule, doxazosin, activates EphA2 in a ligand-dependent manner and suppresses tumor cell metastasis in prostate cancer^56^. In addition to the possible ligand-independent crosstalk between EphA2 and Akt, crosstalk between EphA2, growth factor receptors, and other intracellular signaling molecules has also been documented^54^. The possible functional linkages between EphA2 and EGFR, MET, or FAK in the promotion of tumor cell migration and proliferation were observed both in vitro and in vivo^54^.

In the present study, we provide a functional pathway-based overview of responses to sorafenib treatment in resistant HCC cells and a systematic method of searching for resistance-related therapeutic targets. Our results indicate that sorafenib-mediated protein tyrosine kinase inhibition may promote the activation of alternative cellular pathways such as the Akt, mTOR, and FAK signaling pathways. Furthermore, a novel upstream therapeutic target, EphA2, was identified for future application in the management of sorafenib resistance. The ligand mimic lead compound prazosin was also shown to modulate EphA2 activity. Taken together, our findings provide novel insights into the mechanisms underlying sorafenib resistance in HCC, which may be applied in the development of a rational strategy to block growth and metastatic progression and reduce the spread of tumors.

## Supplementary information

Supplemental figures and legends
