# Supplementary material for: Quantitative phosphoproteomic analysis identifies the potential therapeutic target EphA2 for overcoming sorafenib resistance in hepatocellular carcinoma cells
Source: Exp Mol Med. 2020 Mar 19;52(3):497–513. doi: 10.1038/s12276-020-0404-2 (PMC7156679; doi:10.1038/s12276-020-0404-2)

**Supplemental figure legends**

**Supplemental Fig 1. Screening of different hepatoma cell lines for sorafenib sensitivity and EphA2 expression levels.** (a) All five cell lines (HuH-7, HuH-7^R^, PLC-5, Sk-Hep-1 and Hep3B) was growth to 90% confluence for 24 hr and harvested. Total EphA2 and p-EphA2 S897 expression levels were determined via western blotting with the indicated antibodies. (b) Five HCC cell lines, HuH-7, HuH-7^R^, PLC-5, Sk-Hep-1 and Hep3B was examined for sorafenib sensitivity by MTT assay after being exposed to sorafenib at the indicated concentrations for 72 hr. The concentration-response curve for sorafenib was fit to each cell lines.

**Supplemental Fig 2. EphA2 mediates proliferation, migration, invasion and sorafenib sensitivity in Sk-Hep-1 cells.** (a) Sk-Hep-1 cells were infected with lentiviruses containing shEphA2 (#1, #2) or control shRNA (shCtrl), and 48 hr later, lysed and analyzed via western blotting with the indicated antibodies. (b) Viability of EphA2 knockdown Sk-Hep-1 cells was determined at the indicated time-points with the MTT assay. Plots depict cumulative cell numbers versus days in culture. (c) Wound healing assay of shEphA2-infected Sk-Hep-1 cells. The micrographs show cells that migrated into the gap 0 h and 24 hr after removal of the insert. (d) Transwell migration (upper) and invasion (lower) assay of shEphA2-infected Sk-Hep-1 cells. Cells in the central field of each insert were visualized via light microscopy. (f) shCtrl and shEphA2-containing Sk-Hep-1 cells were exposed to sorafenib at the indicated concentrations for 72 hr, and cell viability analyzed with the MTT assay. The concentration-response curve for sorafenib in the EphA2 knockdown group shifted towards a lower concentration, compared to that for shCtrl-infected Sk-Hep-1 cells. All results were representatives from three independent replicates. (**, p < 0.01, ***, p < 0.001, shCtrl, control shRNA; shEphA2, shRNA against EphA2)

**Supplemental Fig 3. EphA2 S897 phosphorylation is important for proliferation, migration, invasion and sorafenib sensitivity in HuH-7^R^ cells.** (a) HuH-7^R^ cells were infected with lentiviruses containing EphA2 S897A mutant or EphA2 wild type (WT) overexpression vectors, and 48 hr later, lysed and analyzed via western blotting with the indicated antibodies. (b) Viability of EphA2 wild type and mutant overexpressed HuH-7^R^ cells was determined at the indicated time-points with the MTT assay. Plots depict cumulative cell numbers versus days in culture. (c) Wound healing assay of EphA2-overexpressed HuH-7^R^ cells. The micrographs show cells that migrated into the gap 0 h and 24 hr after removal of the insert. (d) Transwell migration (upper) and invasion (lower) assay of EphA2-overexpressed HuH-7^R^ cells. Cells in the central field of each insert were visualized via light microscopy. (f) Wild type and mutant EphA2-containing HuH-7^R^ cells were exposed to sorafenib at the indicated concentrations for 72 hr, and cell viability analyzed with the MTT assay. The concentration-response curve for sorafenib in the EphA2 mutant (S897A) group shifted towards a lower concentration, compared to that for EphA2 wild type HuH-7^R^ cells. All results were representatives from three independent replicates. (***, p < 0.001)

**Supplemental Fig 4. Prazosin acts like a ligand-mimic agonist toward EphA2** (a) Simulated Hydrogen bond connections between prazosin and EphA2 LBD. The amino acids, which had the highest probability of hydrogen bond connecting with prazosin, are Ser 68, Val 69, Thr 101, Arg 103, Cys 188, and Val 189. The connections of hydrogen bonds were shown in green dash line, and each percentage of connection time in total 100 ns MD simulation was indicated. (b) Prazosin induced EphA2 internalization in HuH-7^R^ cells. HuH-7^R^ cells were treated with 10 μM prazosin for 0, 2, 4 hr and EphA2 localization was examined by immunofluorescence staining. Cell nuclei were stained with DAPI (blue) and EphA2 were stained with TRITC (red). (c) Inhibitory effects of prazosin on EphA2 and Akt phosphorylation in Sk-Hep-1 cells. Sk-Hep-1 cells were treated with 10 μM prazosin for the indicated times. Cell lysates were separated via SDS-PAGE and analyzed using western blotting with the specified antibodies.


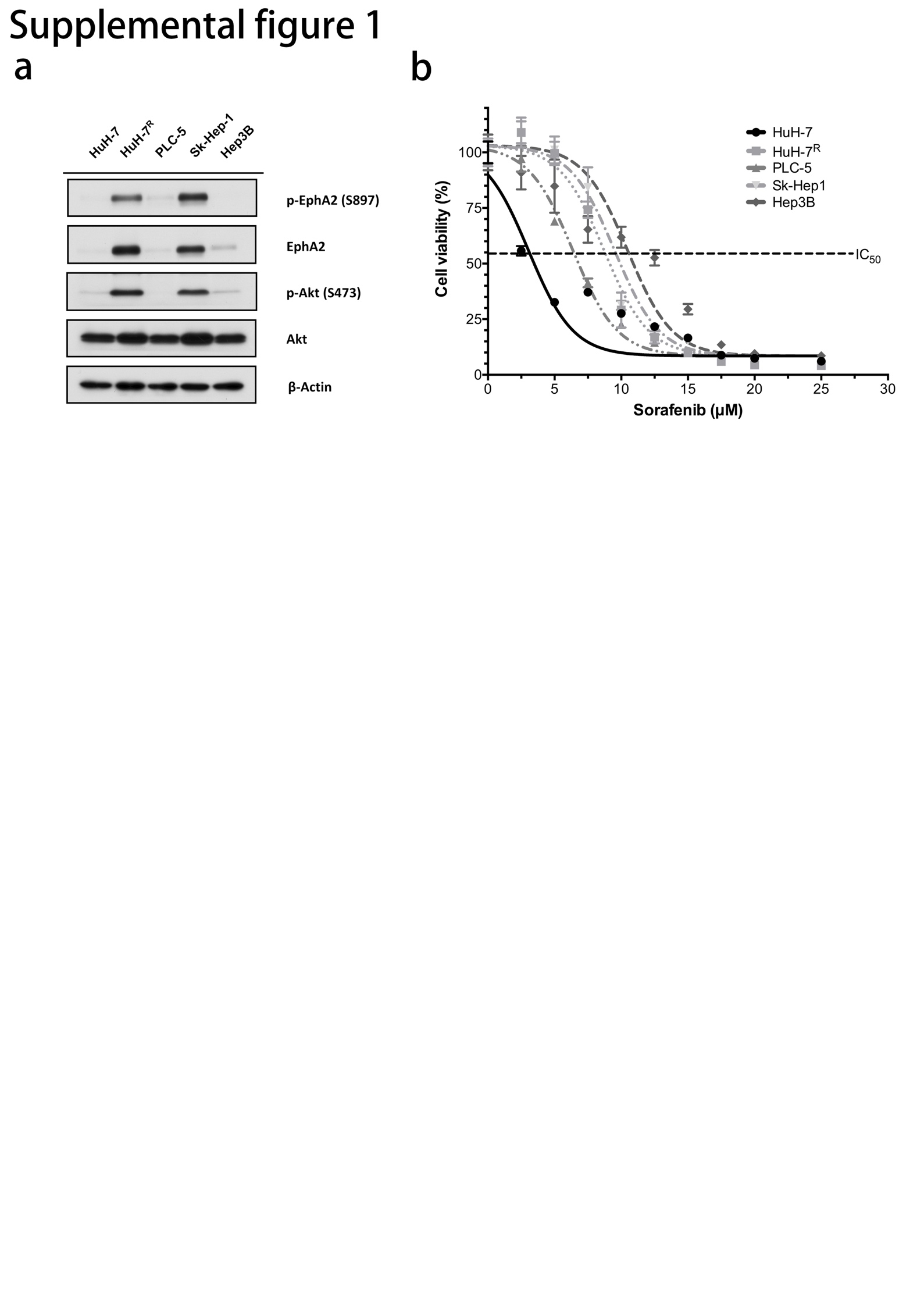


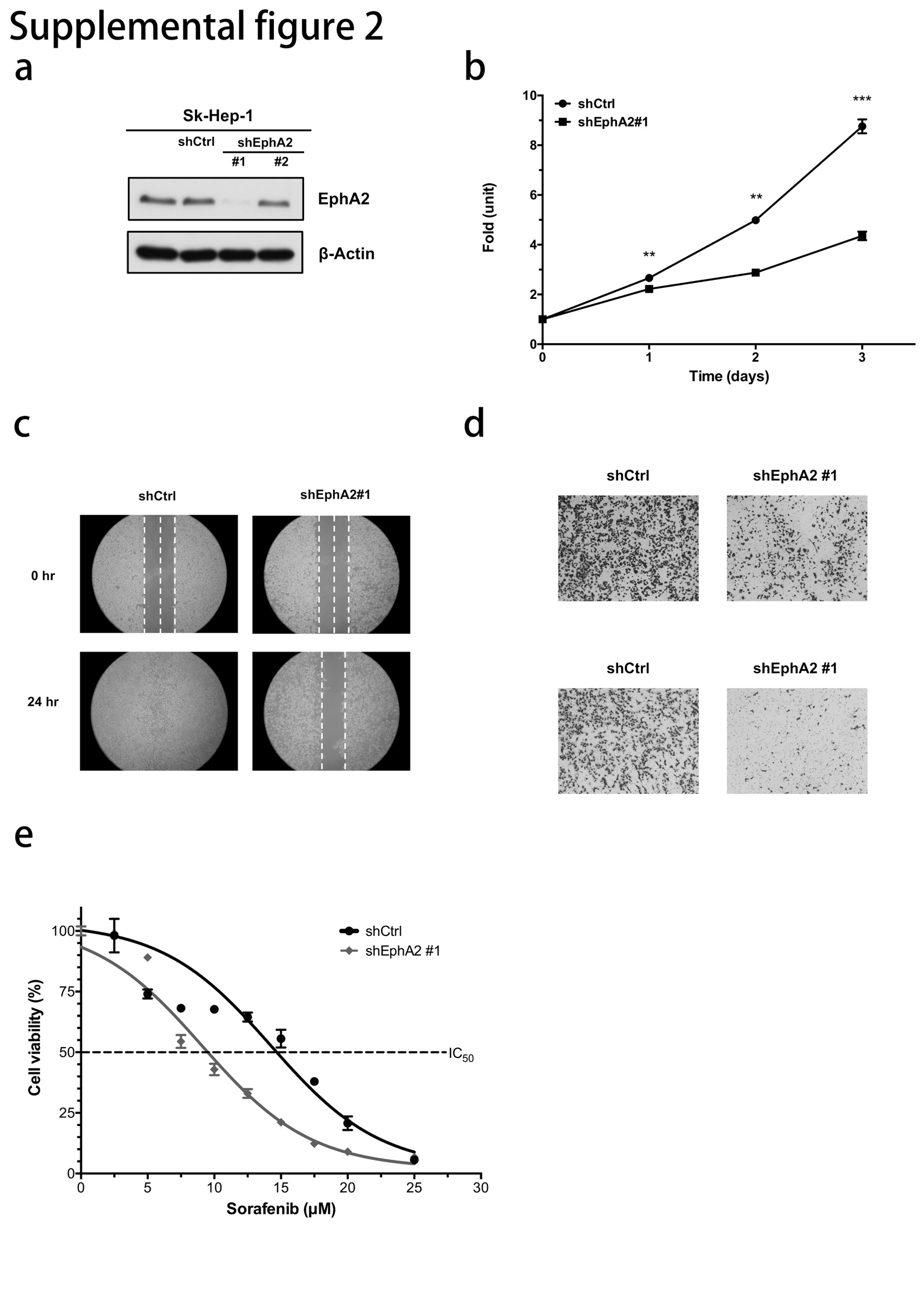


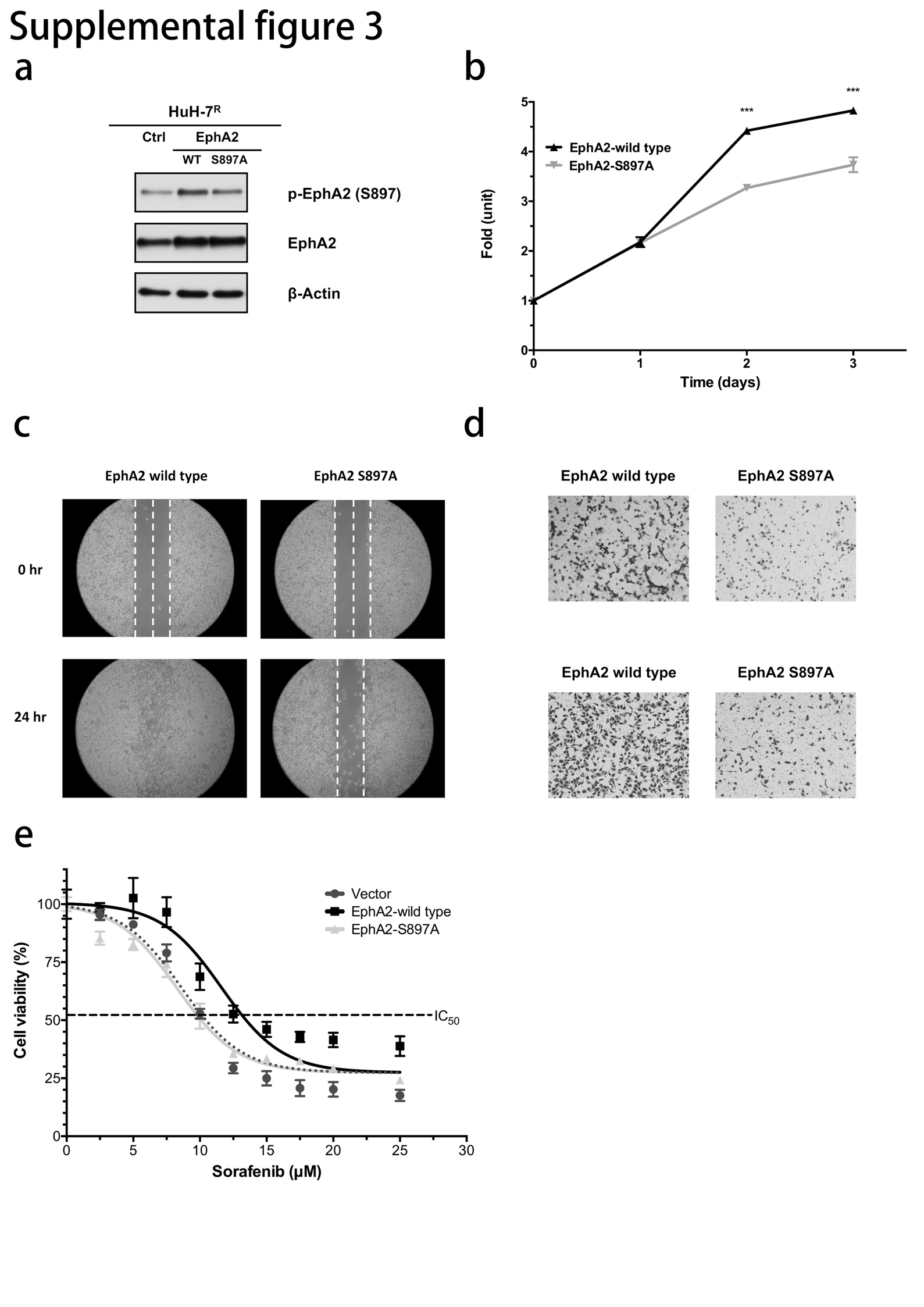


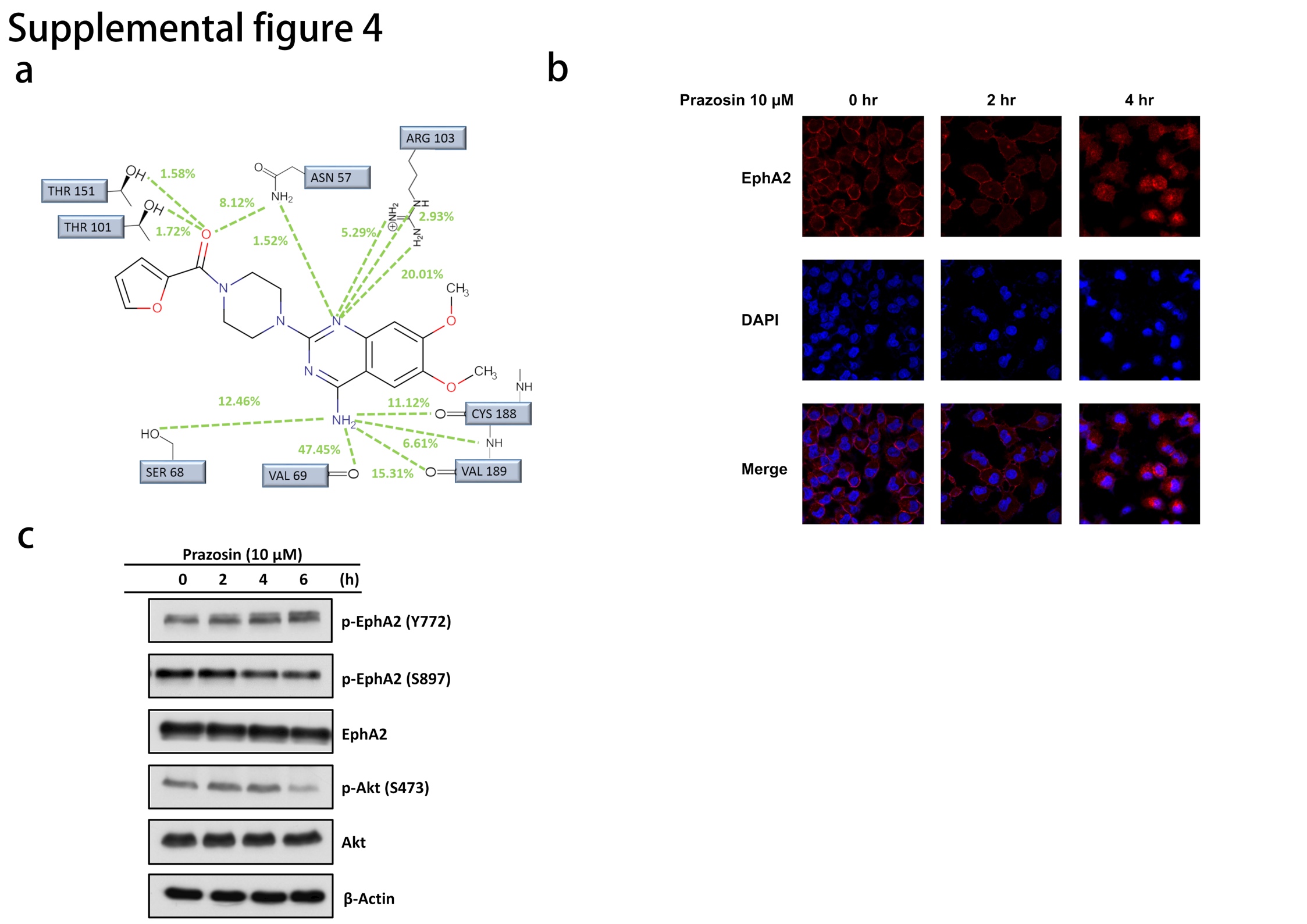

Supplement: Supplementary file 1 — Supplemental figures and legends [file 12276_2020_404_MOESM1_ESM.docx]
